# **Erratum to:** 2021 ISHNE / HRS / EHRA / APHRS Collaborative Statement on mHealth in Arrhythmia Management: Digital Medical Tools for Heart Rhythm Professionals: From the International Society for Holter and Noninvasive Electrocardiology / Heart Rhythm Society / European Heart Rhythm Association / Asia Pacific Heart Rhythm Society

**DOI:** 10.1093/ehjdh/ztab086

**Published:** 2021-11-08

**Authors:** Niraj Varma, Iwona Cygankiewicz, Mintu Turakhia, Hein Heidbuchel, Yufeng Hu, Lin Yee Chen, Jean‐Philippe Couderc, Edmond M Cronin, Jerry D Estep, Lars Grieten, Deirdre A Lane, Reena Mehra, Alex Page, Rod Passman, Jonathan Piccini, Ewa Piotrowicz, Ryszard Piotrowicz, Pyotr G Platonov, Antonio Luiz Ribeiro, Robert E Rich, Andrea M Russo, David Slotwiner, Jonathan S Steinberg, Emma Svennberg

https://doi.org/10.1093/ehjdh/ztab001

In the originally published version of this manuscript, disclosure of several authors’ handling editor statuses was omitted from the supplementary material in error. This error has been corrected online.

