# Corrigendum to: 2021 ISHNE / HRS / EHRA / APHRS Collaborative Statement on mHealth in Arrhythmia Management: Digital Medical Tools for Heart Rhythm Professionals: From the International Society for Holter and Noninvasive Electrocardiology / Heart Rhythm Society / European Heart Rhythm Association / Asia Pacific Heart Rhythm Society

**DOI:** 10.1093/ehjdh/ztac060

**Published:** 2022-11-01

This is a corrigendum to: Niraj Varma, Iwona Cygankiewicz, Mintu Turakhia, Hein Heidbuchel, Yufeng Hu, Lin Yee Chen, Jean-Philippe Couderc, Edmond M Cronin, Jerry D Estep, Lars Grieten, Deirdre A Lane, Reena Mehra, Alex Page, Rod Passman, Jonathan Piccini, Ewa Piotrowicz, Ryszard Piotrowicz, Pyotr G Platonov, Antonio Luiz Ribeiro, Robert E Rich, Andrea M Russo, David Slotwiner, Jonathan S Steinberg, Emma Svennberg, 2021 ISHNE / HRS / EHRA / APHRS Collaborative Statement on mHealth in Arrhythmia Management: Digital Medical Tools for Heart Rhythm Professionals: From the International Society for Holter and Noninvasive Electrocardiology / Heart Rhythm Society / European Heart Rhythm Association / Asia Pacific Heart Rhythm Society, European Heart Journal - Digital Health, Volume 2, Issue 1, March 2021, Pages 7–48, https://doi.org/10.1093/ehjdh/ztab001

In the originally published version of this manuscript, the data availability statement was accidentally omitted.

This error has now been corrected.

