# Supplementary material for: 2021 ISHNE / HRS / EHRA / APHRS Collaborative Statement on mHealth in Arrhythmia Management: Digital Medical Tools for Heart Rhythm Professionals: From the International Society for Holter and Noninvasive Electrocardiology / Heart Rhythm Society / European Heart Rhythm Association / Asia Pacific Heart Rhythm Society
Source: Eur Heart J Digit Health. 2021 Jan 29;2(1):7–48. doi: 10.1093/ehjdh/ztab001 (PMC9708018; doi:10.1093/ehjdh/ztab001)
Supplement: ztab001_Supplementary_Data [file ztab001_supplementary_data.docx]

**Author disclosure table**

| **Writing group member** | **Employment** | **Honoraria/ Speaking/ Consulting** | **Speakers’ bureau** | **Research*** | **Fellowship support*** | **Ownership/ Partnership/Principal/**  **Majority stockholder** | **Stock or stock options** | **Intellectual property/ Royalties** | **Other** |
| --- | --- | --- | --- | --- | --- | --- | --- | --- | --- |
| Niraj Varma, MA, MD, PhD, FACC, FRCP (Chair) | Cleveland Clinic, Cleveland, Ohio | None relevant | None | None | None | None | None | None | International Editor at Annals of Noninvasive Electrocardiology |
| Iwona Cygankiewicz, MD, PhD (Vice-Chair) | Medical University of Lodz, Lodz, Poland | None | None | None | None | None | None | None | 0: ISHNE (President, 2019-2021) |
| Mintu P. Turakhia, MD, MS, FHRS (HRS Vice-Chair) | Center for Digital  Health, Stanford University, Stanford,  California, and VA  Palo Alto Health  Care System, Palo  Alto, California | 1: Pfizer, Inc.; 3: Medtronic; 3: AliveCor; 4: BIOTRONIK; 4: Abbott | None | 5: American Heart  Association; 5: Apple; 5: AstraZeneca; 5: Boehringer  Ingelheim; 5: Bristol-Myers Squibb;  5: Janssen  Pharmaceuticals | None | None | None | None | None |
| Hein Heidbuchel, MD, PhD, FESC, FEHRA (EHRA Vice-Chair) | Antwerp University and University Hospital, Antwerp, Belgium | None | None | None | None | None | None | None | None |
| Yufeng Hu, PhD (APHRS Vice-Chair) | Taipei Veterans General Hospital, Taipei City, Taiwan | None | None | None | None | None | None | None | None |
| Lin Yee Chen, MD, FHRS | University of Minnesota, Minneapolis, Minnesota | None | None | NIH grants: R01HL141288 and R01 HL126637 | None | None | None | None | None |
| Jean-Phillippe Couderc, MBA, PhD | University of Rochester, New York | ERT | None | None | None | VPG Medical, Inc.; ERT | None | None | Section Editor (Bioengineering) at Annals of Noninvasive Cardiology |
| Edmond M. Cronin, MBChB, FHRS, CCDS, CEPS-A | Temple University, Philadelphia, Pennsylvania | None | None | None | None | None | None | None | None |
| Jerry Estep, MD | Cleveland Clinic, Cleveland, Ohio | Medtronic; Abbott; Newpulse; Getinge group | None | None | None | None | None | None | None |
| Lars Grieten, PhD, MSc | Hasselt University, Hasselt, Belgium | None | None | None | None | 0: FibriCheck | None | None | None |
| Deirdre A. Lane, PhD, FHEA, FESC | Institute of Cardiovascular Sciences, University of Birmingham, Birmingham, United Kingdom | Bristol-Myers Squibb /Pfizer, Boehringer Ingelheim, and Daiichi-Sankyo | None | None | None | None | None | None | None |
| Reena Mehra, MD | Cleveland Clinic; Cleveland, Ohio | None | None | NIHLBI [U01HL125177, UG3HL140144]; AHA | None | None | None | None | None |
| Alex Page PhD | Neurology Dept.  University of Rochester | None | None | None | None | None | None | None | None |
| Rod S. Passman, MD, FHRS | Northwestern University, Evanston, Illinois | 1: Medtronic; 1: Janssen Pharmaceuticals  Abbott | None | AHA Foundation;  Pfizer; Medtronic | None | None | None | 2: UptoDate | None |
| Jonathan P. Piccini, Sr., MD, MHS, FHRS | Duke University Medical Center, Durham, North Carolina | Abbott, Allergan, ARCA Biopharma, Biotronik, Boston Scientific, LivaNova, Medtronic, Milestone, Myokardia, Sanofi, Philips | None | Abbott, Association for the Advancement of Medical Instrumentation, Bayer, Boston Scientific, and Philips | None | None | None | None | None |
| Ewa Piotrowicz, MD, PhD | Cardinal Stefan Wyszynski National Institute of Cardiology, Warsaw, Poland | None | None | None | None | None | None | None | None |
| Ryszard Piotrowicz, MD, PhD | Cardinal Stefan Wyszynski National Institute of Cardiology, Warsaw, Poland | None | None | None | None | None | None | None | None |
| Pyotr G. Platonov, MD, PhD, FHRS | Lund University, Lund, Sweden | None | None | None | None | None | None | None | Associate Editor at Annals of Noninvasive Cardiology |
| Antonio Luiz Ribeiro, MD, PhD | Universidade Federal de Minas Gerais, Belo Horizonte, Brazil | None | None | None | None | None | None | None | Dr Ribeiro is supported in part by CNPq (310679/2016-8 and 465518/2014-1) and by FAPEMIG (PPM-00428-17). |
| Robert Rich  BA, MBA, PhD (Hon) | Chairman - Rich Products Corp.;  Chairman Emeritus - Cleveland Clinic | None | None | None | None | None | None | None | None |
| Andrea M. Russo, MD, FHRS, FACC | Cooper Medical School of Rowan University, Camden, New Jersey | None | None | 1: MediLynx; 2: Boehringer Ingelheim; 2: Boston Scientific | None | None | None | 1: UpToDate | 0: ABIM (Member, ABIM Cardiovascular Board); 0: ABMS (Membership on Advisory Committee or Review Panels); 0: Apple Inc. (Steering Committee Apple Heart Study); 0: Boston Scientific (Steering Committee, Research) |
| David J. Slotwiner, MD, FHRS | NewYork-Presbyterian Queens, Flushing, New York, and Cardiology Division, Weill  Cornell Medical College, New York, New York | None | None | None | None | None | None | None | Section Editor for Cardiovascular Digital Health Journal |
| Jonathan S. Steinberg, MD, FHRS | University of Rochester, Rochester, New York | ABIM; Allergan; AtriCure, Inc.; Corfigo; Medtronic; National Cardiac; Omron | None | National Institutes of Health | None | None | AliveCor | None | Section Editor (Defibrilation/Resynchronization) at Annals of Noninvasive Cardiology |
| Emma Svennberg, MD | Karolinska Institutet Danderyds Hospital, Solna, Sweden | 1: Bayer Healthcare Pharmaceuticals; 1: Boehringer Ingelheim; 1: Bristol-Myers Squibb; 1: Merck; 1: Pfizer, Inc.; 1: Sanofi | None | 4: Roche Diagnostics | None | None | None | None | None |

NIH: National institute of health; AHA = American Heart Association.
